# Influence of Pelvic Intensity-Modulated Radiation Therapy With Concurrent Cisplatin-Based Chemotherapy of Cervical Cancer on the Vaginal Microbiome

**DOI:** 10.3389/fonc.2021.615439

**Published:** 2021-02-23

**Authors:** Li Jiang, Bo Li, Yong Zhang, Shanshan Ma, Chang Liu, Feifei Liang, Zhuxin Wei, Tingting Huang, Rensheng Wang

**Affiliations:** Department of Radiation Oncology, The First Affiliated Hospital of Guangxi Medical University, Nanning, China

**Keywords:** vaginal microbiome, cervical cancer, intensity-modulated radiation therapy, radiochemotherapy, 16S rRNA sequencing

## Abstract

Pelvic intensity-modulated radiation therapy (IMRT) combined with concurrent chemotherapy is an effective treatment for cervical cancer; however, radiation resistance impairs its clinical benefit. The vaginal microbiome plays an important but poorly understood role in cancer radiochemotherapy. In this study, we investigated the effects of treatment on the overall composition and alteration of the vaginal microbiome in patients receiving pelvic IMRT with concurrent cisplatin-based chemotherapy. We collected samples from twenty patients with cervical cancer and six healthy controls and performed 16S rRNA sequencing. Vaginal microbial composition analysis revealed significant differences between the two groups, but no significant differences between radiation treatment time points. However, the relative abundances of *Gammaproteobacteria, Gemmatimonadetes, Gemmatimonadales, Pseudomonadales, Gemmatimonadaceae, Rikenellaceae, Acinetobacter, Desulfovibrio, Prevotella 9, Rikenellaceae RC9 gut group, Turicibacter*, and the *metagenome* increased with time. The results encourage further study into the effects of the vaginal microbiome on cervical cancer treatment strategies, especially radiochemotherapy. Better understanding of these effects could inform new therapeutic approaches to enhance the efficacy of radiochemotherapy.

## Introduction

Cervical cancer, the most common gynecological malignancy, occurs in the epithelial lining of the cervix (1). For patients with locally advanced cervical cancer, radiotherapy combined with chemotherapy has become the mainstream treatment, usually involving intensity-modulated radiation therapy (IMRT) (2, 3). Although IMRT provides high dose conformity and spares organs at risk, resistance to treatment is an obstacle for patients with cervical cancer (4). Therefore, strategies to enhance the effects of radiation and chemotherapy are required to obtain better clinical outcomes.

The human microbiome is the collection of microorganisms that inhabit the mucosal surfaces of the body, including the vagina (5). In recent years, sequencing technology has made great progress in cataloguing these populations, with 16S ribosomal RNA (rRNA) sequencing most commonly used. Changes in the vaginal microbiome (VM) are associated with cervical cancer development (6–8), and evidence is rapidly mounting that it can affect cancer treatment outcomes through diverse mechanisms (9, 10). The VM can therefore be considered a novel target to improve the treatment sensitivity of cervical cancer. However, the role of the VM in patients with cervical cancer who receive pelvic IMRT combined with chemotherapy is not well understood. To assess the effects of pelvic radiochemotherapy on the VM, we compared the VM profiles of patients with cervical cancer and healthy controls, and then tracked the changes to the VM in patients with cervical cancer during pelvic IMRT combined with concurrent cisplatin-based chemotherapy by bacterial 16S rRNA gene sequencing.

## Methods

### Patients and Treatment

A prospective study was conducted by enrolling 20 patients with cervical cancer who received radical radiochemotherapy at the First Affiliated Hospital of Guangxi Medical University from April 2016 to May 2017. The inclusion criteria were: 1) patients were scheduled for pelvic IMRT at a dose delivered to a planning clinical target volume (PCTV) of 50 Gy in combination with concomitant cisplatin-based chemotherapy, 2) availability for vaginal sampling using a sterile swab stick, and 3) willingness to participate. The exclusion criteria were: 1) recent treatment with antibiotics, steroids, or immune-suppressants, 2) distant metastasis, and 3) previous pelvic radiotherapy for another tumor or with palliative intent. In addition, six healthy individuals were also enrolled from April 2016 to May 2017. Their inclusion criteria were 1) no history of malignancy, 2) a Karnofsky performance status ≥ 90, and 3) willingness to undergo vaginal sampling using a sterile swab stick. Written informed consent was obtained from all subjects, and the research protocol was approved by the Ethical Review Committee of the First Affiliated Hospital of Guangxi Medical University. Information that can be used to identify individual participants during or after the data collection is available and can be accessed. We confirm that all methods were performed in accordance with relevant guidelines and regulations.

All patients underwent a contrast-enhanced CT scan in the supine position with an immobilization device. The images datasets were imported to the treatment planning system (TPS). The gross tumor volume (GTV) and clinical target volume (CTV) was determined by CT and MRI. The CTV of the tumor bed (CTV-T) included the range of 10 mm from GTV as well as the entire uterus, cervix, parametria, and at least 3 cm proximal of the vagina. The nodal CTV (CTV-N) was delineated to include bilateral iliac, obturator, and presacral lymphatic drainage region with an expansion of the blood vessels by 7 mm. The planning clinical target volume (PCTV) was generated by a uniform expansion of 10 mm from CTV-T and 7 mm from CTV-N. The PCTV was prescribed such that > 95% was received at ≥ 50 Gy in 25 fractions, five times weekly for 5 weeks. The pelvic IMRT plan was generated using seven-field beam. In addition, all patients received weekly brachytherapy at a dose of 30 to 35 Gy following the pelvic IMRT. The constraints for organs at risk (OARs) were defined according to the institutional guidelines. Concurrent cisplatin-based chemotherapy was adopted in conjunction with IMRT as part of the treatment protocol. The concurrent chemotherapy regimen comprised the administration of cisplatin alone (80–100 mg/m^2^) every 3 weeks for two cycles.

### Sampling and DNA Extraction

A sterile swab stick was used to obtain a specimen from the cervical lesion using aseptic technique. When possible, four sequential samples were collected based on radiation treatment time points: before starting pelvic radiotherapy (the first time point sample, T1), after the fifth radiotherapy session (the second time point sample, T2), after the 15th radiotherapy session (the third time point sample, T3), and after the 25th radiotherapy session (the fourth time point sample, T4). T1 samples were obtained 1 week before starting radiotherapy. The swab tops were placed in 2-ml sterile DNAase/RNase-free cryovials containing phosphate-buffered saline (400 μl), and stored at −80°C until further processing. A cell lysis procedure including enzymatic lysis and bead beating was used prior to DNA extraction using a QIAamp DNA Mini Kit (Qiagen, Hilden, Germany) and amplification by polymerase chain reaction (PCR).

### Library Preparation and Sequencing

In total, 71 vaginal swabs with sufficiently high-quality DNA were collected. The V3-V4 hypervariable regions of the 16S rRNA gene were amplified with the primers 338F (5′-ACTCCTACGGGAGGCAGCA-3′) and 806R (5′-GGACTACHVGGGTWTCTAAT-3′) on a 2720 Thermal Cycler (Applied Biosystems, USA) (11, 12). PCR was conducted using the following program: 2 min at 98°C, then 20 cycles of 30 s at 98°C, 30 s at 50°C, and 1 min at 72°C, followed by a final incubation at 72°C for 5 min. Reactions were performed in triplicate. The reaction mix (50 μl total) consisted of 2×TransStart FastPfu Fly PCR SuperMix (25 μl), each primer (1 μl of 10 μM), nuclease-free water (20 μl), and template DNA (10 ng). The resultant PCR products were purified using VAHTS DNA Clean Beads. Secondary PCR was performed under different conditions using special index primers. The PCR program was: 30 s at 98°C, then 10 cycles of 10 s at 98°C, 30 s at 65°C, and 30 s at 72°C, then a final 5 min incubation at 72°C. PCR reactions were performed in triplicate. The reaction mix (50 μl total) consisted of Phusion DNA Polymerase (25 μl), i5/i7 index primers (1 μl of 2.5 μM), UltraPure water (13 μl), and purified PCR product (10 μl). Reactions were analyzed on 1.8% agarose gels to ensure successful amplification. Unsuccessful reactions were repeated after a 10× dilution of the initial template concentration, and removed from the experiment if unsuccessful again. PCR products were extracted from the agarose gels, further purified using an AxyPrep DNA Gel Extraction Kit (Axygen Biosciences, USA), and eluted with Tris-HCl. ImageJ (National Institutes of Health, Bethesda, MD, USA) was used to quantify the electrophoresis results. The library was pooled at equimolar concentrations and resolved on a 1.8% agarose gel, and the 600 bp band was extracted. The purified library was paired-end sequenced (2 × 250) on an Illumina HiSeq platform (Illumina, San Diego, CA, USA) according to the manufacturer’s instructions.

### Bioinformatic Analysis

FLASH (1.2.11) was used to merge paired end reads with a minimum overlap of 10 bp (13). Primer and barcode sequences were trimmed using cutadapt (v1.18) (14); and chimeric sequences were detected and removed with VSEARCH (v2.13.6) (15). The trimmed data were processed to form OTUs at 97% identity using VSEARCH, and a representative OTU was selected from each cluster (15, 16). Using the Silva_132 16S rRNA database as a reference, Ribosomal Database Project classifiers were used to assign taxonomic ranks to each OTU using Qiime (v1.9.1) (16, 17). The alpha-diversity and beta-diversity indices were calculated based on the rarefied OTU counts. Alpha-diversity was performed in Mothur (v1.38.1) (18, 19), and represents an analysis of the diversity in a single sample, reflected by parameters including the Sobs, Chao1, Ace, Shannon, and Simpson indices. The Wilcoxon rank-sum test was used to compare alpha-diversity indices. Beta-diversity is a measure of the microbiota structure between groups. Both weighted and unweighted UniFrac distance matrices were plotted in the PCA, and ANOSIMs were performed using the R package “ade4” (20). For taxa with a prevalence > 10%, differential abundance analysis was performed using the Wilcoxon rank-sum test at the phylum, class, order, family, and genus levels. For multiple comparisons of bacterial counts, the false discovery rate was calculated using the Benjamini-Hochberg method (21). Microorganism features used to distinguish gut microbiotas specific to cervical cancer were identified using the linear discriminant analysis effect size method, with an alpha cutoff of 0.05 and an effect size cutoff of 2.0 (22). Phylogenetic Investigation of Communities by Reconstruction of Unobserved States was used to predict the abundances of functional categories in Kyoto Encyclopedia of Genes and Genomes (KEGG) orthologs (23). Graphing of KEGG pathways at levels 2 (41 pathways) and 3 (328 pathways) was performed with STAMP, and *p* values were calculated using White’s non-parametric t-test *(* 24).

### Statistics

R software (ver. 3.5.1, the R Project for Statistical Computing) was used for statistical analysis. In descriptive analyses, the mean ± standard deviation (s.d.) was used for normally distributed continuous variables and the median ± interquartile range (IQR) was used for continuous variables with skewed distributions. Comparisons of the relative abundance of detected genera between groups were conducted using the Wilcoxon rank-sum test. The Sobs, ACE, Simpson, Shannon, and Chao1 indices were compared using Student’s *t*-test. *P*<0.05 was considered statistically significant.

## Results

### Patient Characteristics

We analyzed 71 vaginal swab samples from 20 patients with cervical cancer and six healthy controls. **Table 1** shows the clinical characteristics of the patients with cervical cancer. Their median age was 54 years (range: 44–73). All patients received pelvic IMRT plus brachytherapy combined with cisplatin-based chemotherapy. Each radiation plan met the prescribed dose requirements, and the mean dose of the planning clinical target volume (PCTV) was calculated as 54 Gy. All 20 patients provided samples at T1, and 15/20 permitted the collection of samples at all four timepoints (T1–T4).

**Table 1:** Clinical characteristics of the patients with cervical cancer (*n* = 20).

Patient characteristic	Number (%)
Age (years)	
≤ 54	10 (50.0%)
>54	10 (50.0%)
FIGO^a^ classification	
I B2	1 (5.0%)
II A2	3 (15.0%)
II B	8 (40.0%)
III B	4 (20.0%)
IV A	3 (15.0%)
V B	1 (5.0%)
Histology	
Squamous cell carcinoma	19 (95.0%)
Adenocarcinoma	1 (5.0%)
HPV^b^ status	
Negative	2 (10.0%)
HPV 16	9 (45.0%)
HPV 18	6 (30.0%)
HPV 52	1 (5.0%)
HPV 58	1 (5.0%)
HPV 59	1 (5.0%)
Treatment	
Pelvic IMRT^c^+BT^d^+CT^e^	20 (100.0%)
Patients with four sequential samples (T1–T4)	15 (75.0%)

^a^International Federation of Gynecology and Obstetrics; ^b^human papillomavirus; ^c^intensity-modulated radiation therapy ^d^brachytherapy; ^e^concurrent cisplatin-based chemotherapy.

### VM Diversity Estimations in Patients With Cervical Cancer and Healthy Controls

**Figure 1:**
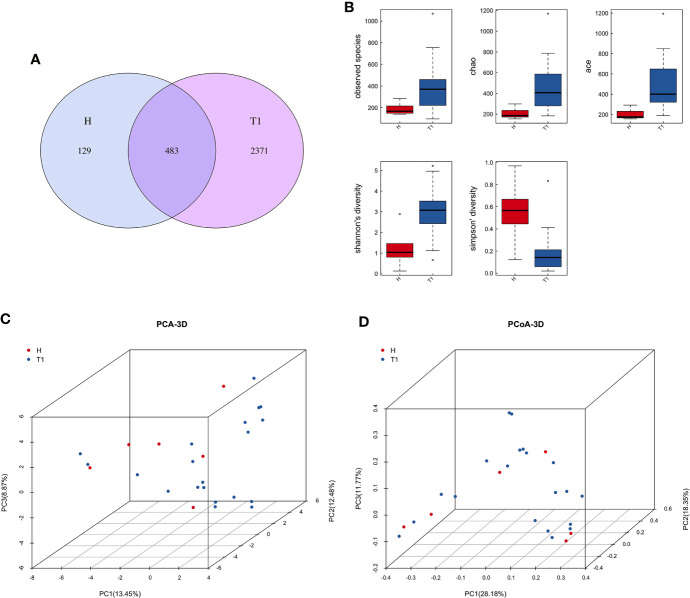
**(A)** Venn diagram for the integration between healthy controls and cervix cancer patients OTUs. **(B)** Principal component analysis (PCA) of vaginal microbiome of 20 patients and 6 healthy controls. **(C)** The comparison between patients and healthy controls by alpha diversity analysis. **(D)** The comparison between patients and healthy controls by beta diversity analysis. H, Healthy controls; T1, the first time point sample of patients.

After quality control processes and the removal of chimeric sequences, we obtained 2,983 operational taxonomic units (OTUs) in total. **Table S1** summarizes the numbers of unique sequences and OTUs in each normalized sample. Of these, 612 OTUs (20.5%) were detected in healthy controls, 2,500 (83.8%) were detected in patients with cervical cancer, and 483 OTUs (16.2%) were detected in both groups (**Figure 1A**). Abundance comparisons of individual OTUs through principal component analysis (PCA) revealed differences in the VM composition of healthy controls and patients with cervical cancer (**Figure 1B**). Furthermore, species diversity and richness were also higher in cervical cancer samples than in control samples. The Chao, Ace, Shannon, and Simpson indices (*p* = 0.016, = 0.002, <0.001, <0.003, and <0.006, respectively) are shown in **Figure 1C** and **Table 2**. Beta diversity analysis revealed statistically significant differences between the two groups (*p* = 0.005; weighted UniFrac and analysis of similarity (ANOSIM); **Figure 1D**).

**Table 2:** The comparison between patients with cervical cancer and healthy controls by alpha diversity analysis.

Alpha diversity	Mean (H)	s.d. (H)	Mean (T1)	s.d. (T1)	*P* value
Sobs	186.83	54.44	387.05	224.61	0.016
Chao	204.84	53.14	460.55	236.70	0.002
Ace	199.61	52.64	492.35	253.55	0.0001
Shannon	1.23	0.93	3.06	1.14	0.003
Simpson	0.56	0.28	0.19	0.19	0.006

T1, the first time point sample of patients; H, healthy controls; s.d., standard deviation.

### VM Diversity Estimations in Patients With Cervical Cancer During Treatment

**Figure 2:**
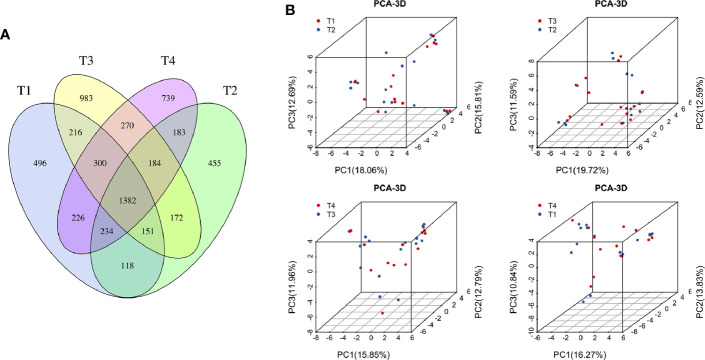
**(A)** Venn diagram for the integration among the four time points of patient samples. **(B)** 3D scatter plot of PCA results for comparison among four time points of patient samples (T1 vs T2, T3 vs T2, T4 vs T3, and T4 vs T1). T1, the first time point sample; T2, the second time point sample; T3, the third time point sample; T4, the fourth time point sample.

**Figure 3:**
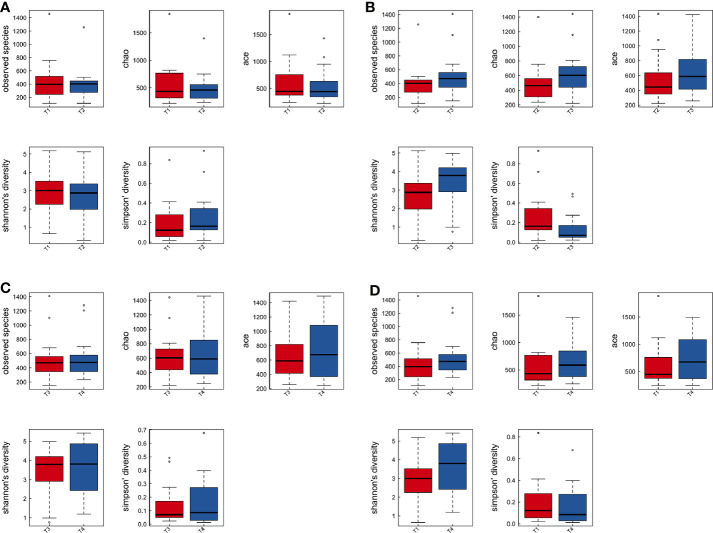
The comparison among the four time points of patient samples by alpha diversity analysis. Boxplots of alpha diversity in T1 vs T2 **(A)**, T3 vs T2 **(B)**, T4 vs T3 **(C)**, and T4 vs T1 **(D)** using different metrics (observed OTUs, the Shannon index, and the Simpson index). T1, the first time point sample; T2, the second time point sample; T3, the third time point sample; T4, the fourth time point sample.

To explore the effects of radiation on the VM during the treatment process, we first analyzed VM richness and diversity at four time points (T1–T4). In total, 6,109 OTUs were obtained, and 1,382 were common among all timepoints. Then, the bacterial compositions at each timepoint were compared using overlap analysis (**Figure 2A**) and 3D-PCA (**Figure 2B**). Interestingly, in comparisons of T1 vs T2, T3 vs T2, T4 vs T3 and T4 vs T1, the timepoints were not spatially distinct, nor were they significantly different in VM richness and diversity according to alpha (**Figures 3A–D**) and beta diversity analyses (data not shown).

### Impact of Radiation Therapy on the VM Composition of Patients With Cervical Cancer

**Figure 4:**
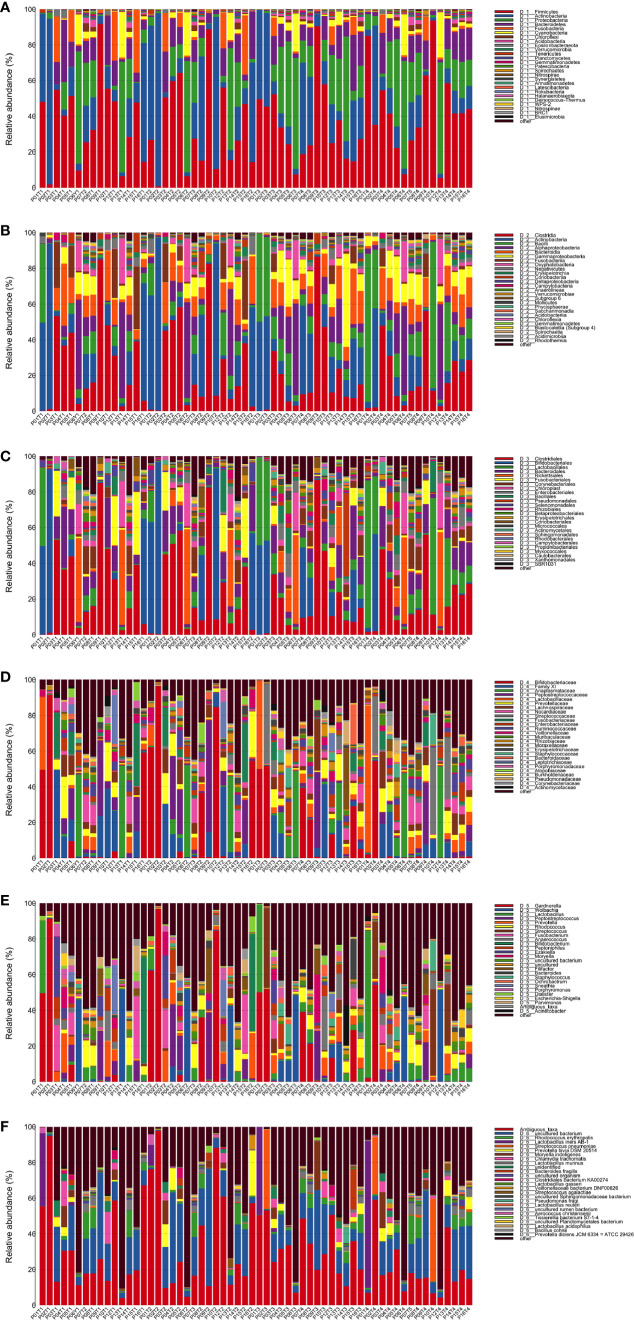
Distribution of bacterial phyla **(A)**, classes **(B)**, orders **(C)**, families **(D)**, genera **(E)**, and species **(F)** obtained by next-generation sequencing of samples from 15 patients at four time points.

**Figure 5:**
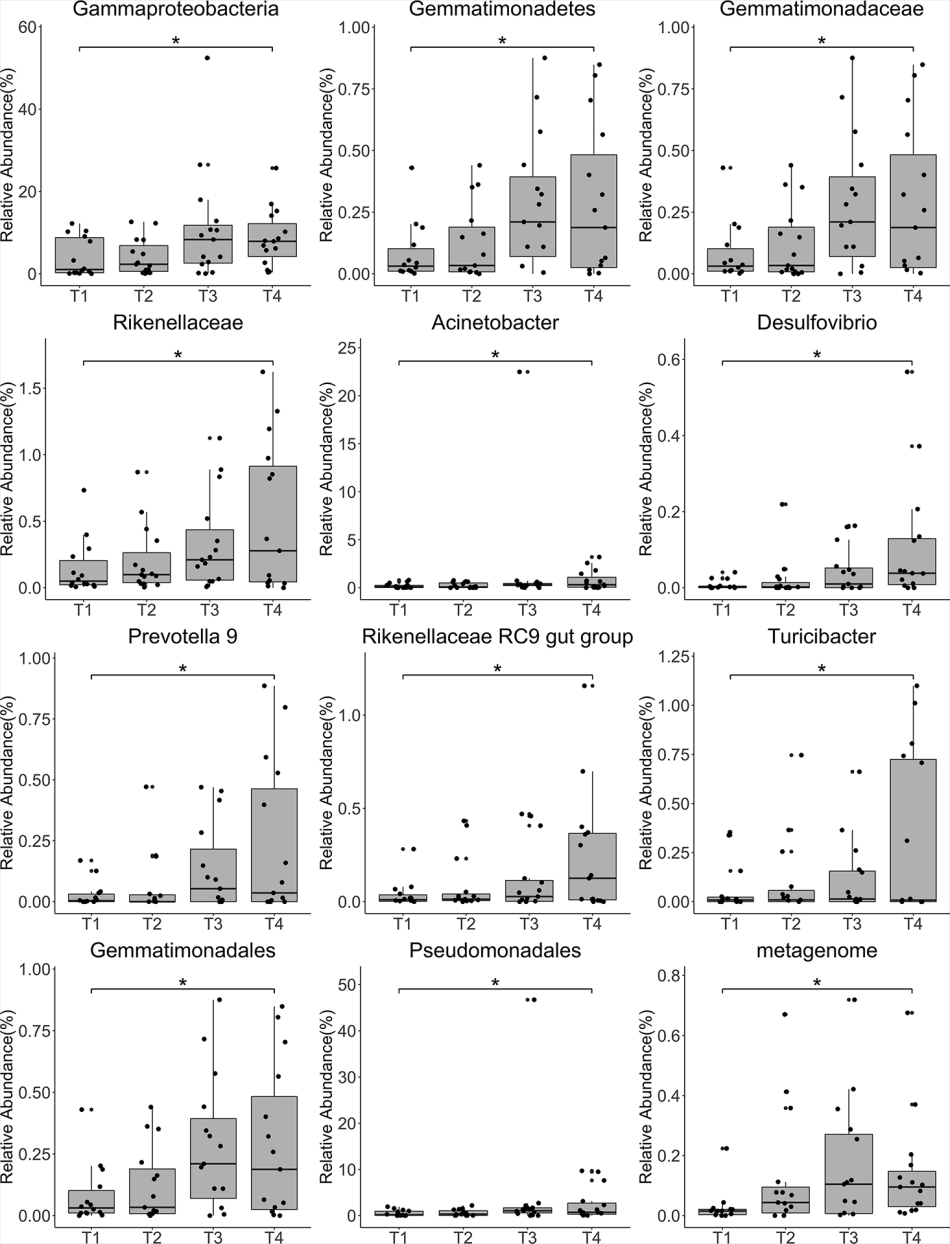
Relative abundances of the top 12 most abundant genera at each time point. Each bar represents the mean abundance ± the standard error of the mean. *P<0.05.

Annotation analysis revealed the distribution of the microbiota at the phylum, class, order, family, genus, and species levels over time (**Figures 4A–F**). **Figure 5** shows changes in the relative abundances of major phylum-level taxa during radiation therapy. The relative abundances of Gammaproteobacteria, Gemmatimonadetes, Gemmatimonadales, Pseudomonadales, Gemmatimonadaceae, Rikenellaceae, Acinetobacter, *Desulfovibrio*, *Prevotella* 9, the Rikenellaceae RC9 gut group, *Turicibacter*, and the metagenome increased with radiation time.

## Discussion

Cervical cancer often results from persistent infection with human papillomavirus (HPV), which induces cervical epithelial cells to become cancerous (25). However, the process of cervical carcinogenesis can be affected by the VM (8), and increasing evidence indicates that VM changes play important roles in the process (26, 27). Conversely, cervical cancer disrupts the ratio between commensal and pathogenic microbiome species, resulting in microenvironmental changes (28). However, studies investigating the role of the VM in patients with cervical cancer who receive pelvic radiochemotherapy are scarce. The aim of our study was to examine associations between the use of pelvic radiochemotherapy and VM changes.

Currently, radiochemotherapy is a common treatment strategy for cervical cancer. The National Comprehensive Cancer Network guidelines (V1.2020) for cervical cancer recommend a dose of 45–50 Gy in standard fractionation with IMRT (29). In this study, all patients were prescribed a PCTV of 50 Gy. However, due to limitations in the radiation technique, the dose distribution in the target area is not absolutely uniform. To ensure quality radiation therapy, the patient received at least 50 Gy in 25 fractions, meaning that > 50 Gy was present in the PCTV. Dose data were recorded using a Varian Eclipse V8.0 treatment planning system. IMRT protects organs at risk better than two-dimensional radiation techniques; therefore, our results are based on pelvic IMRT combined with cisplatin-based concurrent chemotherapy.

In our study, there were more OTUs in patients with cervical cancer than in healthy controls, and the abundances of individual OTUs were significantly different between the two groups. When compared at different radiation treatment time points, there are no significant differences found in VM richness and diversity by alpha and beta diversity analysis. Nevertheless, changes in the relative abundances of several taxa were observed during radiation therapy. The dose distribution of radiation is closely associated to the volume and the location of tumor, which might affect distribution of vaginal microbiome during the radiation treatment. The research endpoint may be insufficient to prove the vaginal microbiome as a key clinical index for cervical cancer patients, so a larger cohort is needed to determine the correlation between VM and cervical cancer patients in future study.

It is well established that VM imbalance is strongly correlated with cervical cancer. Vaginal dysbiosis (characterized by a non-*Lactobacillus*-dominant composition) and inflammation have been associated with HPV persistence and progression to cervical cancer (30). Compared to patients diagnosed with low− and high-grade squamous intraepithelial lesions, increased levels of *Lactobacillus crispatus*, *Lactobacillus iners*, and *Lactobacillus taiwanensis* were observed in the vaginal swabs of healthy women, while *Gardnerella vaginalis* and *Lactobacillus acidophilus* were absent. The bacterial dysbiosis observed in these patients, which featured a predominance of *G. vaginalis* and a concomitant paucity of *L. crispatus*, *L. iners*, and *L. taiwanensis*, may be associated with the development of HPV-dependent cervical cancer (31). However, whether pelvic IMRT affects the vaginal microbiome remains unknown.

A previous study compared differences in the proportions of bacteria isolated before and after radiotherapy using aerobic culture. The results showed no significant changes in the positive cultures of pathogens; however, the normal flora significantly increased after external beam irradiation (32). However, methods based on next-generation sequencing (NGS) were not used in this study. Using 16S rRNA sequencing, we observed no significant changes in overall diversity before, during, and after radiotherapy, consistent with the above study. However, the relative abundances of several taxa, including *Gammaproteobacteria, Pseudomonadales, Gemmatimonadaceae*, and *Prevotell*a 9, increased significantly with irradiation time.

The tumor microenvironment can have great impact on radioresistance and tumor recurrence (33). The microbiome can also affect cancer by triggering DNA damage, modulating inflammation, and generating metabolites (10). Several previous studies have shown that patients receiving radiotherapy display obvious changes in the microbiomes of the irradiated areas as well as changes in the microenvironment, indicating that the microbiome may serve as an aberrant proinflammatory factor. A study by Wang *et al*. showed that *Gammaproteobacteria, Pseudomonadales*, and *Prevotella 9*, which showed increased abundance with radiation time in this study, were more abundant in the fecal microbiome after pelvic radiation, and were strongly associated with diagnoses of radiation enteritis. *In vitro* experiments indicated that radiation-induced microbiome dysbiosis results in epithelial cell damage, promoting inflammatory responses in the local mucosa by activating nuclear factor κB (NFκB) signaling and cytokine secretion (34). Radiation can reduce resistance to commonly used antibiotics, and vancomycin pretreatment can enhance the antitumor effects of radiation *in vivo* by increasing antigen presentation and cytotoxic T cell infiltration into the tumor, through modulation of the gut microbiota (32, 35). It has been suggested that microbiome superantigens might promote radiotherapy-induced inflammation by activating T cells and attenuating epithelial cell recovery (36). Increasing evidence indicates that inflammatory signaling pathways such as the toll-like receptor/myeloid differentiation primary response 88, proinflammatory cytokine, NFκB, and cyclooxygenase-2 pathways are bridging factors between the microbiome and cancer (37). The detection of *Gemmatimonadaceae* DNA in the blood has been associated with tumor progression in patients treated with nivolumab (38). While some studies have found no benefit to probiotic use (39), a meta-analysis demonstrated a beneficial effect of probiotics in reducing the incidence of diarrhea (40). Furthermore, one study reported that the anaerobic bacteria *Clostridium novyi*-NT, which is missing its major toxin gene, is able to selectively destroy the hypoxic regions of tumors and improve the efficacy of radiation in mouse tumor models (41).

In conclusion, we conducted a bioinformatic analysis of the VM in patients with cervical cancer receiving pelvic radiochemotherapy and healthy controls by 16S rRNA sequencing. We firstly examined microbiome differences between the cervical cancer patients and health controls, and then investigated the impact of pelvic radiochemotherapy on the VM in patients with cervical cancer. Our data indicated some changes in the relative abundances of the microbiome, which might have critical effects on the efficacy of radiochemotherapy. Future studies will be required to understand the relationship between the VM and radiochemotherapy in cervical cancer.

## Data Availability Statement

The data sets presented in this study can be found in online repositories. The names of the repository/repositories and accession number(s) can be found below: 16S rRNA squencing data to SRA (https://www.ncbi.nlm.nih.gov/sra/). The SRA ID is PRJNA687644.

## Ethics Statement

The studies involving human participants were reviewed and approved by The Ethical Review Committee of the First Affiliated Hospital of Guangxi Medical University. The patients/participants provided their written informed consent to participate in this study.

## Author Contributions

RW and TH designed the study. YZ and SM collected samples and followed up the patients. BL, FL, and CL performed the experiments. LJ and ZW performed the data analyses. LJ wrote the main manuscript text and BL prepared all figures and tables. RW and TH critically inspected the manuscript and participated in its revision. LJ and BL contributed equally to this study and are the co-first authors of this paper. All authors contributed to the article and approved the submitted version.

## Funding

This study was supported by the Youth Science Foundation of Guangxi Medical University (GXMUYSF201505), the Basic Ability Enhancement Project for Young Teachers in Guangxi Zhuang Autonomous Region (2018KY0134), the Guangxi Zhuang Autonomous Region Health and Wellness Committee Science and Technology Project (S2017017), the Key Research and Development Program of Guangxi (Guike AB18281003), the “139” Program for high-level medical talents in Guangxi, Innovation Team of the First Affiliated Hospital of Guangxi Medical University, and the Guangxi Science and Technology Program Project (GK AD17129013). The funders had no role in study design, data collection and analysis, decision to publish, or preparation of the manuscript.

## Conflict of Interest

The authors declare that the research was conducted in the absence of any commercial or financial relationships that could be construed as a potential conflict of interest.

## Supplementary Material

Supplementary Table 1 The OTUs of cervical cancer patients and healthy controls. CV, cervical cancer patients; HC, healthy controls.
