# Supplementary material for: Influence of Pelvic Intensity-Modulated Radiation Therapy With Concurrent Cisplatin-Based Chemotherapy of Cervical Cancer on the Vaginal Microbiome
Source: Front Oncol. 2021 Feb 23;11:615439. doi: 10.3389/fonc.2021.615439 (PMC7940522; doi:10.3389/fonc.2021.615439)
Supplement: Supplementary Table 1 — The OTUs of cervical cancer patients and healthy controls. CV, cervical cancer patients; HC, healthy controls. [file Table_1.docx]

Table S1 The OTUs of cervical cancer patients and healthy controls.

| **Sample Name** | **Tag number** | **OTU number** |
| --- | --- | --- |
| CV 01 | 70022 | 138 |
| CV 02 | 48143 | 217 |
| CV 03 | 19168 | 224 |
| CV 04 | 58374 | 96 |
| CV 05 | 39334 | 253 |
| CV 06 | 47269 | 142 |
| CV 07 | 64052 | 754 |
| CV 08 | 42417 | 530 |
| CV 09 | 80330 | 518 |
| CV 10 | 67007 | 365 |
| CV 11 | 29972 | 369 |
| CV 12 | 84816 | 363 |
| CV 13 | 87068 | 1068 |
| CV 14 | 14768 | 425 |
| CV 15 | 84668 | 371 |
| CV 16 | 27873 | 384 |
| CV 17 | 41868 | 386 |
| CV 18 | 24677 | 454 |
| CV 19 | 35851 | 466 |
| CV 20 | 69698 | 218 |
| HC 01 | 71383 | 284 |
| HC 02 | 88103 | 215 |
| HC 03 | 46599 | 174 |
| HC 04 | 86457 | 140 |
| HC 05 | 21898 | 160 |
| HC 06 | 55305 | 148 |

CV, cervical cancer patients; HC, healthy controls.
